# Screening for Alpha 1 antitrypsin deficiency in Tunisian subjects with obstructive lung disease: a feasibility report

**DOI:** 10.1186/1750-1172-4-12

**Published:** 2009-04-15

**Authors:** Sabri Denden, Michele Zorzetto, Fethi Amri, Jalel Knani, Stefania Ottaviani, Roberta Scabini, Marina Gorrini, Ilaria Ferrarotti, Ilaria Campo, Jemni Ben Chibani, Amel Haj Khelil, Maurizio Luisetti

**Affiliations:** 1 Biochemistry and Molecular Biology Laboratory, Faculty of Pharmacy, AV. Avicienne 1, 5019 Monastir, Tunisia; 2 Center for Diagnosis of Inherited Alpha1-antirtypsin Deficiency, Institute for Respiratory Disease, IRCCS San Matteo Hospital Foundation, Piazza Golgi 19, 27100 Pavia, Italy; 3 Pediatric Department, Ibn El Jazzar Hospital, Av Ibn El Jazzar, 3100 Kairouan, Tunisia; 4 Pulmonology Department, CHU Tahar Sfar, 5111 Mahdia, Tunisia

## Abstract

**Background:**

AATD is one of the most common inherited disorders in the World. However, it is generally accepted that AATD in North African populations is not a risk factor for lung and/or liver disease, based on a number of small studies. We therefore planned a screening study for detection of AATD in patients with OLD in a cohort of patients from Kairouan in central Tunisia. Methods: One hundred twenty patients with OLD (asthma, emphysema, COPD) were enrolled in the screening programme. Laboratory diagnosis for AATD was performed according to current diagnostic standards.

**Results:**

We found that 6/120 OLD patients carried an AAT deficient allele, 1 PI*MZ, 1 PI*MPlowel, 3 PI*MMmalton, 1 PI*MMwurzburg.

**Conclusion:**

this pilot study demonstrated that alleles related to deficiency of AAT are not absent in the Tunisian population, and that rare AATD variants prevailed over commonest PI*Z variant. These results would support a larger scale screening for AATD in Tunisia.

## Background

Alpha 1 antitrypsin (AAT) is an acute phase glycoprotein predominantly derived from the liver, and its major biological function is to inhibit neutrophil elastase [1]. AAT, a highly polymorphic protein with more than 120 variants known to date [2] is coded by a gene, called *SERPINA1*, located on chromosome 14q31-32.3 within the SERPIN cluster [3]. The *SERPINA1 *PI*M alleles code for the commonest normal AAT variants, whereas PI*S and PI*Z are the most common deficiency alleles associated with reduced concentrations of plasma AAT. Nevertheless, there are at least 30 *SERPINA1 *alleles rarely detected, other than the PI*Z and the PI*S alleles, which are associated with significantly reduced AAT levels [4-6].

Inherited deficiency of AAT (AATD) is one of the most common genetic disorders in the world, and is associated with an increased risk of developing lung and, to a lesser extent, liver disease [7]. Individuals homozygous for the PI*Z allele usually have an AAT level in the region of 0.35 g/L [3] and are at high risk for the development of emphysema [8], asthma [9], chronic bronchitis and bronchiectasis [10]. However, heterozygous individuals with PI*S and PI*Z mutation (or rare mutations) showing a more protective AAT level (> 0,8 g/L) [3] can also be at increased risk for lung disease, depending on multiple environmental factors, such as smoking, occupational exposure, and environmental exposure.

Epidemiology studies showed that highest prevalence of PI*ZZ related AATD is recorded among Northern Europeans and populations with North European background [11]. Notwithstanding, during the last few years, based on estimates from allele frequencies obtained in available cohort studies, it has been suggested that the Z variant is not only common in Caucasians, but also among other ethnic groups worldwide [12,13].

A total of 30 cohorts have been investigated for AATD in the African continent. Twenty four cohorts, having a total of 4,718 individuals, were in Sub-Saharian Africa [14], 6 cohorts with a total sample size of 1,735 have been investigated in the north African populations [15-20], Three of them in Tunisia: in the Tunisian population, the PI*Z allele, previously considered as virtually absent [15,16], has then been detected once on the heterozygous state in a total cohort of 1,168 individuals (allele frequency 0.04%) [17]. However, none of the previous surveys in Tunisia has looked at rare, non-S and non-Z SERPINA1 variants, which have been hypothesised to be particularly frequent in the Mediterrenean area, where PI*Z allele frequency is reduced [4]. We therefore aimed this paper at investigating AATD variants in a Tunisia area, targeting a cohort of obstructed individuals, in which the diagnosis of AATD has been recommended accoding to the ATS/ERS document [21].

## Methods

### Study subjects

Upon approval by the local Ethical Committee, patients with obstructive lung disease (OLD: asthma, emphysema, COPD), referred to the pulmonary disease department in Kairouan regional hospital (central Tunisia) from June 2006 to September 2006, were enrolled in the screening programme. Diagnosis of obstructive lung disease was obtained by symptoms, radiology, lung function examinations and allergy history.

### Quantitative determination of AAT level

AAT concentration measurement was performed on plasma samples with a rate immune turbidimetric method (Konelab 20, Thermo Clinical Labsystem, Finland), using a polyclonal anti-human AAT antibody (Thermo Electron Corporation, Finland). A calibrator (Specical, Thermo Electron Corporation, Finland) with an assigned AAT value was used as a standard. A control (Specitrol, Thermo Electron Corporation, Finland) with an assigned AAT value was used after a predefined reaction number. Plasma samples for the turbidimetric assay are diluted by the instrument 1:10 (AAT reading range, 0.23–4.4 g/L). The turbidimetry of antigen-antibody complex was measured at 360 nm. Values obtained are regressed according to the calibration curve, and expressed as g/L.

### Molecular diagnosis of AATD

DNA extracted from the white blood cells of all subjects by standard methods, was submitted to genotyping for Z and S *SERPINA1 *alleles, by PCR-RFLP, as previously described [22]. According to published diagnostic algorythm [23], samples negative for Z and S alleles, but with inconsistent AAT level/genotype were submitted to sequencing of *SERPINA1*exons II, III, IV, and V, as previously described [24].

## Results

**Figure 1:**
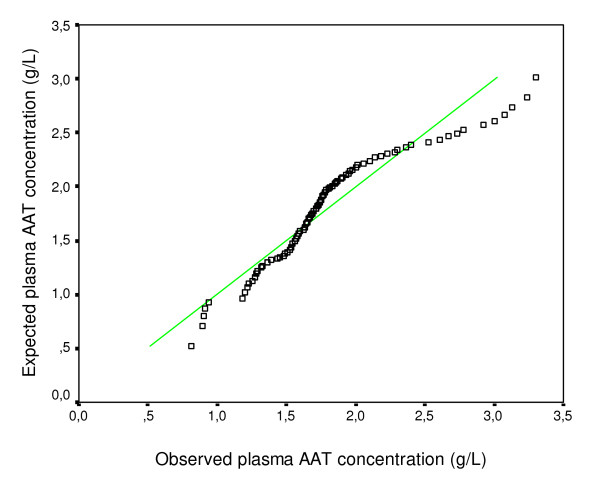
**AAT plasma level distribution in OLD patients**. The expected normal values (in green) were calculated using Rankit proportional estimation.

One hundred twenty subjects with OLD were enroled in this study. There were 115 males and 5 females, mean age (SD) 49.2 (9.5) years (range 34–81). The large majority of patients were smokers (84.1%). As far as the OLD phenotype was concerned, there were 49 COPD (40.8%), 48 emphysema (40%), and 23 asthma patients (19.2%). Mean (SD) plasma AAT level was 1.74 g/L (0.63) (range 0.81 to 3.3). Plasma AAT level distribution is shown in figure 1.

*SERPINA1 *S and Z allele genotyping, performed in all OLD subjects, allowed the detection of one PI*MZ individual (plasma AAT level: 0.81 g/l, consistent with the detected genotype).

**Figure 2:**
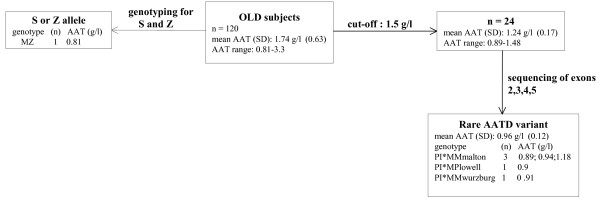
**Schematic representation of the genotyping/sequencing results**.

Nevertheless, some subjects negative for PI*S and PI*Z allele detection, displayed plasma AAT levels consistent with intermediate AATD; in particular, 4 subjects displayed plasma AAT level < 1 g/L [mean (SD) 0.91 g/L (0.02)]. According to our protocol for detection of rare AATD variants, such samples with inconsistent AAT level/genotype were suitable for sequencing [4,24]. In the absence of reference values of plasma AAT in the general population from Tunisia, we decided to submit to sequencing, DNA samples from subjects with plasma AAT levels < 1.5 g/L, rather than the usual cut-off of 1.13 g/L [25]. We therefore sequenced 24 DNA samples (plasma AAT level range: 0.89–1.48). We found that 5 subjects were heterozygous for rare deficiency variants: 3 subjects carried the PI* MMmalton, 1 the PI*MPlowell, and 1 the PI*MMwurzburg genotypes. Figure 2 summarises the results of genotyping/sequencing.

## Discussion

Meta-analysis of available data showed that *SERPINA 1 *PI*S and PI*Z deficiency alleles are extremely rare in North African populations [12,13]. Recent investigations in the Tunisian population confirmed the low frequency of AATD common deficiency variants in the general population and healthy subjects (1078 subjects investigated: 15 PI*MS detected (PI*S: 0.7%; PI*Z: 0%)) [15-17], as well as in COPD patients (90 subjects investigated: 1 PI*SS and 1 PI*MZ detected (PI*S: 1%; PI*Z: 0.5%)) [17]. The present study, performed in 120 patients with OLD, confirms these data: PI*S allele was not seen, whereas PI*Z was detected only once on the heterozygous state (frequency 0.4%).

In this study, we extended the *SERPINA1 *gene investigation to variants not detectable by rapid PI*S and PI*Z genotyping: by this strategy, we found 5 more deficiency variants (frequency as a whole 2%) detected on the heterozygous state. Such a condition of a relatively higher frequency of rare than common AATD variants, is shared by Central – Southern regions of Italy, in which PI*Mmalton and PI*Mprocida variants seem to prevail over PI*Z [4]. Interestingly, the PI*Mmalton variant, detected in three unrelated individuals in this paper, is the commonest AATD variant in Sardinia, where the PI*Z variant is detected very rarely [4,26]. A novel *SERPINA1 *Null mutation, first described in an Egyptian family, and for that reason called Q0*cairo [27] has been repeatedly detected in unrelated individuals from regions of Southern Italy. Population admixture due to migration occurred in ancestral periods, as well as contacts for commercial purposes in more recent centuries are likely to be responsible for dissemination of rare AATD variants in the Southern Mediterrenean basin. Table 1 summarizes the rare variants reported in the Mediterranean basin, as well as the mutations types, the cellular defect and the related clinical data [4,28-30].

**Table 1:** Rare AATD variants reported in the Mediterranean basin, type of mutation involved, cellular defect and the related clinical data.

**PI allele**	**Mutation type**	**Cellular defect**	**Associated disease**
M_malton_	3 bp deletion	intracellular aggregation	lung, liver
M_procida_	1 bp substitution	intracellular degradation	lung
P_lowell_	1 bp substitution	intracellular degradation	lung
I	1 bp substitution	intracellular aggregation	lung, liver
M_varallo_	30 bp deletion/22-bp insertion	unkown	lung
M_heerlen_	1 bp substitution	intracellular degradation	lung
M_wurzburg_	1 bp substitution	intracellular aggregation	lung
Q0_isola di procida_	17 kb deletion	no mRNA	lung
Q0_clayton_	1 bp insertion	truncated protein	lung
Q0_cairo_	1 bp substitution	unkown	lung
Y_barcelona_	2 substitutions of 1 bp	unkown	lung
Q0_lisbon_	1 bp substitution	unkown	lung
M_vall d'hebron_	1 bp substitution	unkown	lung

This study strengthens the concept that for a correct laboratory diagnosis of AATD, there is a need of a combination of biochemical and biomolecular methods [24], otherwise rare AATD variants will be missed [5]. These preliminary data also confirm the usefulness of enrolling patients with OLD in a screening programme for AATD. According to the ATS/ERS statement's evidence based recommendations, all subjects with COPD and asthma should be submitted to diagnostic testing for AATD [21]. In this paper, although no subjects with severe AATD deficiency were detected, we found six out of 120 subjects (5%) carrying the so called "intermediate deficiency", that means heterozygosity with one normal PI*M allele and one severe AATD allele [mean (SD) plasma AAT level: 0.93 g/L (0.12)]. These findings are consistent with the hypothesis that intermediate AATD, such as PI*MZ or equivalent genotypes, represent a risk factor for developing COPD [20]. Recent meta-analysis [31] reported the increase in risk of COPD in PI*MZ heterozygous individuals (OR for PI*MZ versus PI*MM (normal genotype) was 2.31 (95% CI 1.60 to 3.35)).

Severe AATD diagnosis in the North African countries might be beneficial to the treatment of patients with the introduction of AAT replacement therapy in these populations. Furthermore, AATD carrier diagnosis might provide genetic counseling to persons who are planning a pregnancy or are in the prenatal period [21]. In addition, awareness of carrying a gene that may increase the susceptibility to COPD may be an additional factor for a successful enrolment of patients in smoking cessation programmes [32].

## Conclusion

This investigation, performed for the first time with current diagnostic standards in a North African population, highlights the implication of AATD in development of OLD in this area, whereas previous data excluded a role for AATD in the African continent. This would suggest that, similarly to what happens in the Caucasians, also in Northern Africans AATD is an underdiagnosed condition, and therefore investigations in larger sample sizes would be advisable. It seems also likely that targeted detection strategy to identify affected individuals produces a rate of detecting disease higher than the population-based screening programs. Such approach may also contribute to change the widespread concept concerning the AAT deficiency epidemiology in North African and others populations.

## Competing interests

The authors declare that they have no competing interests.

## Authors' contributions

SD, MZ, IF and IC designed the study and drafted the Manuscript. FA and JK participated in data collection. SO, RS and MG participated in laboratory investigations. JBC, AHK and ML supervised the study process, revised and edited the final manuscript.
